# Prophylactic ciprofloxacin treatment prevented high mortality, and modified systemic and intestinal immune function in tumour-bearing rats receiving dose-intensive CPT-11 chemotherapy

**DOI:** 10.1038/sj.bjc.6605051

**Published:** 2009-04-28

**Authors:** H Xue, C J Field, M B Sawyer, L A Dieleman, V E Baracos

**Affiliations:** 1 Department of Oncology, University of Alberta, Cross Cancer Institute, 11560 University Avenue, Edmonton, Alberta T6G 1Z2, Canada; 2 Alberta Institute for Human Nutrition, University of Alberta, 4-126A HRIF East, Edmonton, Alberta T6G 2E1, Canada; 3 The Center of Excellence for Gastrointestinal Inflammation and Immunity Research, University of Alberta, 2-24 Zeidler Ledcor Centre, 130 University Campus, Edmonton, Alberta T6G 2X8, Canada

**Keywords:** CPT-11, ciprofloxacin, infectious complication, immunity, gut

## Abstract

Infectious complications are a major cause of morbidity and mortality from dose-intensive cancer chemotherapy. In spite of the importance of intestinal bacteria translocation in these infections, information about the effect of high-dose chemotherapy on gut mucosal immunity is minimal. We studied prophylactic ciprofloxacin (Cipro) treatment on irinotecan (CPT-11) toxicity and host immunity in rats bearing Ward colon tumour. Cipro abolished chemotherapy-related mortality, which was 45% in animals that were not treated with Cipro. Although Cipro reduced body weight loss and muscle wasting, it was unable to prevent severe late-onset diarrhoea. Seven days after CPT-11, splenocytes were unable to proliferate (stimulation index=0.10±0.02) and produce proliferative and inflammatory cytokines (i.e., Interleukin (IL)-2, interferon-*γ* (IFN-*γ*), tumour necrosis factor-*α* (TNF-*α*) IL-1*β*, IL-6) on mitogen stimulation *in vitro* (*P*<0.05 *vs* controls), whereas mesenteric lymph node (MLN) cells showed a hyper-proliferative response and a hyper-production of pro-inflammatory cytokines on mitogen stimulation. This suggests compartmentalised effects by CPT-11 chemotherapy on systemic and intestinal immunity. Cipro normalised the hyper-responsiveness of MLN cells, and in the spleen, it partially restored the proliferative response and normalised depressed production of IL-1*β* and IL-6. Taken together, Cipro prevented infectious challenges associated with immune hypo-responsiveness in systemic immune compartments, and it may also alleviate excessive pro-inflammatory responses mediating local gut injury.

Irinotecan (CPT-11) is a water-soluble, semi-synthetic derivative of camptothecin, an alkaloid isolated from *Camptotheca acuminata*. CPT-11 has emerged as a first-line treatment for colon cancer and has been shown to be effective against other malignancies (Rothenberg, 2001); however, its use is limited by its gastrointestinal (GI) and haematological toxicities (Wiseman and Markham, 1996; Hecht, 1998). Dose-intensive systemic chemotherapy is a prevailing tactic used in oncology. However, it presents a potentially fatal challenge to host immunity. Compromised host immunity and infection is a major cause of chemotherapy morbidity and mortality. CPT-11 based regimens consistently compromise the integrity of the intestinal epithelial lining (Cao *et al*, 1998; Gibson *et al*, 2003), which can lead to infection. Intestinal surfaces and local specialised innate and adaptive defences in GI lymphoid tissues (GALTs) are major defences against invasion by pathogens present in the gut lumen. The gastrointestinal lymphoid tissue comprises phenotypically and functionally distinct B, T and accessory cell sub-populations residing in the gut and in the adjacent mesenteric lymph nodes (MLNs) (Hayday and Viney, 2000; Garside *et al*, 2004). Studies on chemotherapy-related suppression and subsequent reconstitution of immune function have been largely confined to immune compartments in peripheral blood (Harris *et al*, 1976; Henon *et al*, 1992; Busca *et al*, 2003). Although less studied, chemotherapy effects on GALT may be of greater importance in response to agents with a dose-limiting intestinal toxicity, such as CPT-11.

Antibiotic prophylaxis is a common strategy for preventing infections in high-risk neutropenic patients receiving chemotherapy, despite concerns over antibiotic resistance (Gafter-Gvili *et al*, 2005; Leibovici *et al*, 2006). An independent review panel, following an excess number of deaths caused by GI toxicities, recommended that CPT-11-treated patients who have persistent loperamide-resistant diarrhoea be treated with fluoroquinolone antibiotics for 7 days (Rothenberg *et al*, 2001). Fluoroquinolone (i.e., ciprofloxacin, Cipro) prophylactic regimens have been shown to be highly effective against chemotherapy-induced bacteremia from gut-colonising bacteria (Gafter-Gvili *et al*, 2005; Leibovici *et al*, 2006). Increasing evidence suggests that fluoroquinolones may exert immunomodulating effects by altering cytokine production of activated T lymphocytes and enhancing haematopoiesis (Riesbeck and Forsgren, 1994; Dalhoff and Shalit, 2003). However, it remains largely unknown how antibiotic prophylaxis will affect the functional competency of systemic and gut immunity during chemotherapy. In this study, we investigated effects of CPT-11 on the gut and systemic immune competence with and without Cipro. Specifically, we sought to isolate roles of opportunistic bacterial infections in the overall toxicity profile of CPT-11, and to study the effects of Cipro on systemic and intestinal immunity.

## Materials and methods

### Animal use

Animal use was approved by the Institutional Animal Care Committee and conducted in accordance with the Guidelines of the Canadian Council on Animal Care. Female Fisher 344 rats (body weight, 150–180 g), 11–12 weeks of age were obtained from Charles River (St Constant, QC, Canada). Rats were housed two per cage in a temperature (22°C) and light-controlled (12 h light) room; water and food were available for *ad libitum* consumption. One week before the experiment, CPT-11 rats were housed individually in wire-bottom cages. The Ward colorectal carcinoma was provided by Dr Y Rustum, Roswell Park Institute Buffalo, NY, USA (Cao *et al* 1998). Non-necrotic tumour pieces (0.05 g) were transplanted subcutaneously (s.c.) on the flank through trocar under slight isoflurane anaesthesia. CPT-11 was provided by Pfizer as a clinical formulation. Atropine (0.6 g l^−1^) was a clinical formulation. Rats were fed a semi-purified diet as described elsewhere (Xue *et al*, 2007).

### Experimental design

Rats transplanted with tumour were randomised to receive Cipro (*n*=11) or not (*n*=20). Cipro was started 1 week before starting CPT-11 and continued throughout the study. All rats had an *ad libitum* access to sterilised drinking water. Cipro was dissolved in drinking water at 100 mg l^−1^ to provide ∼10 mg kg^−1^ per ^·^day. Cipro solutions were prepared every 2–3 days to ensure activity.

When tumour size reached ∼2 cm^3^, CPT-11 therapy was initiated (daily intravenous injections of 150 mg kg^−1^ per day, for 3 days) (Xue *et al*, 2007). Atropine (1 mg kg^−1^ s.c.) was administered before each CPT-11 dose to alleviate early-onset cholinergic symptoms (Xue *et al*, 2007). The day of the first CPT-11 dose was designated as day 0. Seven days after the last CPT-11 dose (day 9), rats were killed. An additional group (controls, *n*=8) of non-tumour-bearing rats that did not receive CPT-11 or Cipro were killed on day 9.

### Outcomes

#### Diarrhoea

A clinically comparable three-point scale was used in grading diarrhoea (Trifan *et al*, 2002); assessments were made by a researcher blinded to study treatments. Grade 3 diarrhoea incidence was calculated for each rat by counting observations of a particular score(s) out of a total of eight observations between day 3 and day 7 when diarrhoea developed to its full severity (Trifan *et al*, 2002). The area under the curve of diarrhoea score was calculated between day 3 and day 7 (Xue *et al*, 2007).

Rats were killed by CO_2_ asphyxiation. Caecal content, the spleen and MLN were collected aseptically. Tibialis anterior and medial gastrocnemius were collected and weighed. Whole blood harvested, respectively at days 0 (as baseline), 3 and 9 was used for a complete blood count and differential white blood cell (WBC) count performed using the Hemavet instrument (CDC Technologies, Oxford, CT, USA). The *β*-glucuronidase activity of caecal contents was determined as described earlier (Xue *et al*, 2007).

*The spleen and mesenteric lymph node cell phenotype* Immune cells were isolated from MLN as described earlier (Field *et al*, 2006). Isolated cells (200 000 cells per well) were used to determine the cell phenotype using a two-colour direct immunofluorescence (Field *et al*, 2000). Antibodies used were CD3, CD4, CD8, CD25, CD28, CD62L, CD71, CD80, CD45RA (BD Bioscience, Mississauga, ON, Canada) and OX12 (Cedarlane, Hornby, ON, Canada); Streptavidin QR (Sigma, Oakville, ON, Canada) was added to all biotin-labelled antibodies. The percentage of cells expressing each antibody marker was determined by flow cytometry (FacScan, Becton Dickinson, Sunnyvale, CA, USA) (Field *et al*, 2000). It was not always possible to perform every phenotype assay on each rat because of the variation in total yield of tissue; *n* for each assay is indicated in result tables.

*Mitogen-induced proliferation and cytokine production* Cells (1.25 × 10^9^ l^−1^) were incubated in a 96-well microtitre plate, in triplicate, in the presence or absence of 5 mg l^−1^ of Concanavalin A (Con A) (ICN, Montreal, PQ, Canada) for 24 and 48 h. Eighteen hours before harvesting, cells were pulsed with 0.5 *μ*Ci of ^3^H-thymidine (Amersham Life Sciences, Baie D’Urfe, PQ, Canada), harvested on glass-fibre paper filters using a multi-well harvester (Skatron, Lier, Norway) and counted in a *β*-counter (LS-5801 Beckman Mississauga, ON, Canada). Proliferation ability was defined as a stimulation index (SI), calculated as ^3^H-thymidine incorporation rates after incubation with Con A/^3^H-thymidine incorporation rates in the absence of Con A.

Splenocytes and MLN cells (1.0 × 10^9^ cells l^−1^) were incubated (48 h) in the presence or absence of lipopolysaccharide (LPS) (100 mg l^−1^) in a 5% v/v CO_2_ humidified atmosphere at 37°C. Supernatants were removed and stored at −70°C until all samples were collected. Interleukin (IL)-1 and -6, interferon-*γ* (IFN-*γ*), tumour necrosis factor-*α* (TNF-*α*) and TGF-*β (*transforming growth factor-*β*) levels were determined using ELISA kits (BD Bioscience) according to the manufacturer’s specifications. Plates were read at 450 nm (SpectraMax 190, Molecular Device, Sunnyvale, CA, USA). Cytokines were assayed in duplicate and variation (co-efficient of variance, CV) between replicates determined. If the CV between duplicates was >15%, samples were re-analysed in duplicate. If a cytokine level was less than the lower detection limit, the half-value of the lower detection limit was used for analysis.

*Bacterial translocation* Mesenteric lymph nodes were aseptically homogenised in 5 ml of sterile water, and 0.1 ml of these samples was inoculated with blood agar (for Gram+ bacteria) and McConkey agar (for Gram– bacteria). Cultures were incubated aerobically at 37°C for 48 h and then colony-forming units on each plate counted and corrected to the original tissue weight.

### Statistics

Data are expressed as mean±s.e.m. Time effects on WBC counts after CPT-11 were analysed through one-way repeated measures analysis of variance (ANOVA) (SPSS 12.0, SPSS Inc., Chicago, IL, USA). Treatment differences in immune phenotypes were analysed using one-way ANOVA followed by *post hoc* Tukey's test, unless specified otherwise. All immune parameters were tested for normal distribution. Values that were not normally distributed were log transformed before statistical analysis. A probability of 0.05 was considered significant.

## Results

### Chemotherapy toxicity

CPT-11 had a 45% mortality rate (9/20) in non-Cipro-treated rats, whereas all rats (11/11) administered Cipro survived. Cipro strikingly improved the overall nutritional status by reducing body weight loss and muscle wasting (Table 1). CPT-11 resulted in diarrhoea of high incidence and cumulative severity, but did not significantly alter diarrhoea profiles (Table 1) or caecal *β*-glucuronidase activity (not shown). CPT-11 resulted in significant bacterial counts in MLN (Gram+ bacteria 3.6±0.7 × 10^3^ CFU per gram tissue and Gram− bacteria 3.5±1.1 × 10^3^ CFU per gram tissue), whereas neither Gram+ nor Gram− bacteria could be detected in the MLN of Cipro-treated rats.

**Figure 1:**
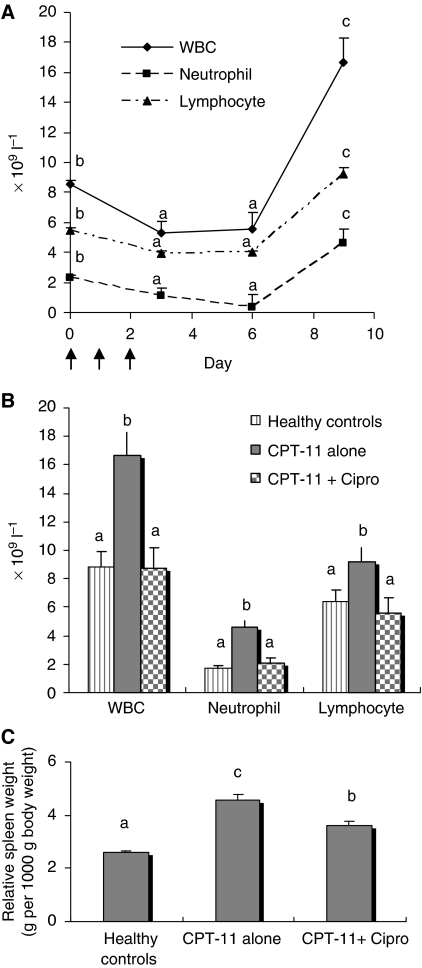
Effects of CPT-11 treatment with or without Cipro on peripheral WBC counts and spleen weight. (**A**) Time course of peripheral WBC counts in rats receiving CPT-11 chemotherapy alone without Cipro. Whole blood was harvested from tumour-bearing rats at the indicated time points after CPT-11. Data (mean±s.e.m.) represent total WBC, neutrophil and lymphocyte counts at corresponding time points. Differences of total WBC, neutrophil and lymphocyte counts at different time points after chemotherapy were analysed by one-way repeated measures ANOVA followed by *post hoc* Tukey's test. Means for a certain count (total WBC, neutrophil or lymphocyte) that do not share a common letter are different, *P*<0.05. (**B**) Differential WBC count in peripheral blood 7 days after CPT-11. Data (mean±s.e.m.) represent total WBC, neutrophil and lymphocyte counts. Means that do not share a common letter are significantly different (*P*<0.05). (**C**) Effects of CPT-11 treatment with or without Cipro on spleen weights. Relative spleen weights (*y* axis) are accounted for the total body weight on day 9. Data are presented as mean±s.e.m. Means that do not share a common letter are significantly different (*P*<0.05).

White blood cell counts and spleen weight of CPT-11 alone led to a transiently depleted peripheral WBC pool, with total WBC, neutrophil and lymphocyte nadirs occurring 1–4 days after completing CPT-11 (Figure 1A). There was a rebound-like recovery of WBC 7 days after completing CPT-11, such that WBC counts (total, neutrophils, lymphoyctes) at this time were significantly higher than at day 0. However, a post-chemotherapy ‘overshoot’ of WBC counts on day 9 was abrogated with Cipro, with no cell counts significantly different from control rats (Figure 1B). Splenic hyperplasia also occurred 7 days after completing CPT-11 and Cipro partially prevented this response (Figure 1C).

### Phenotypic distribution of the spleen and MLN cells

#### Non-Cipro-treated rats

In rats that did not receive Cipro, CPT-11 treatment resulted in a pronounced alteration in the phenotypic composition of immune cells in both the MLN and the spleen (Table 2). Relative effects on T- and B-cell populations in the spleen, with CPT-11, were for the most part similar to those observed in MLN. CPT-11 led to a relative depletion of CD3+ T cells in the spleen (because of a decrease in CD3+CD4+ and CD3+CD8+ populations) and in MLN (because of a decrease in the CD3+CD4+ population) with a relative increase in B-cell proportions (OX12+). Proportions of total cells expressing CD45RA+ (antigen naive marker) were lower in MLN and in the spleen after CPT-11 for a larger percentage of B and CD8+ T cells, but not CD4+ T cells, for which there was a relative increase in antigen-mature (CD45RA−) cell proportions after CPT-11. There was a marked change in the expression of activation markers by T cells after CPT-11. In the spleen and MLN, there was a striking increase (4–13 fold) in helper and suppressor T populations that expressed the co-stimulatory molecule, CD28 (*P*<0.05). Within helper T populations in the spleen and MLN, there were more cells expressing transferrin (CD71+) and IL-2 (CD25+) receptors but less cells expressing L-selectin (CD62L+).

#### Cipro-treated rats

The overall phenotypic changes after CPT-11 were in a similar direction in rats receiving Cipro as compared with that in non-Cipro-treated rats. Nevertheless, Cipro further reduced proportions of CD3+ after CPT-11 in MLN, because of a reduction in proportions of CD3+CD8+ cells. The percentage of B cells was higher in Cipro-treated MLN. Cipro restored proportions of CD8+CD45RA+ in the spleen to proportions not different from that of control rats. In MLN, the lower percentage of CD3+CD8+ cells after Cipro seemed to be because of a lower number of CD8+CD45RA− (antigen mature) cells. As for other T-cell activation markers, the most consistent finding was that Cipro resulted in a strikingly higher number of helper and suppressor T cells expressing IL-2 receptors in MLN (but not the spleen). A higher proportion of total T cells expressing CD28+ with CPT-11 remained unaffected (or even further enhanced in suppressor T cells of MLN) with Cipro in both the MLN and the spleen. In MLN and the spleen, Cipro was associated with a higher proportion of suppressor T cells expressing CD71, but lowered the proportion of CD71+ helper T cells after CPT-11 as compared with non-Cipro-treated rats. Cipro lowered numbers of suppressor T cells expressing L-selectin after CPT-11, as compared with non-Cipro-treated rats.

### Proliferative response to Con A

In CPT-11-treated rats, basal (unstimulated) rates of ^3^H-thymidine uptake by MLN cells (24 and 48 h) and splenocytes (24 h) were significantly higher than in control rats (Table 3). Cipro resulted in an unstimulated rate of ^3^H-thymidine uptake that was comparable or even below control rat levels. CPT-11 completely inhibited splenocyte stimulation in response to Con A, but stimulated responses to Con A in MLN (Table 3). Responses by cells from Cipro-treated rats were not significantly different from controls in MLN, but Cipro did not normalise responses in the spleen.

### Mitogen-stimulated cytokine production of splenocytes and MLN cells

In the absence of mitogen (unstimulated), a spontaneous production of IL-10 and TNF-*α* by MLN cells, and IL-6, TNF-*α* and IFN-*γ* by splenocytes was below detection limits. Only control splenocytes produced detectable amounts of IL-1*β* (64±6 × 10^−9^ g l^−1^) in the absence of mitogens. Splenocytes from Cipro-treated rats produced IL-10 in the absence of mitogen (610±30 × 10^−9^ g l^−1^) producing significantly more (*P*<0.05) than non-Cipro-treated rats (426±39 × 10^−9^ g l^−1^, *P*<0.01) or controls (472±39 × 10^−9^ g l^−1^).

Table 4 summarises the effects of CPT-11 treatment with or without Cipro on mitogen-stimulated cytokine production by splenocytes and MLN cells.

1. Proliferative cytokine, IL-2 Compared with the control group, CPT-11 suppressed IL-2 production by splenocytes in response to both Con A and anti-CD3/CD28 (*P*<0.05), and these effects were not reversed by Cipro treatment. By contrast in MLN, the amount of IL-2 produced after Con A stimulation by the CPT-11 group was higher than in controls (*P*<0.05), and this increase was reversed by Cipro (i.e., Cipro lowered IL-2 production to levels not significantly different from that of controls in MLN).
2. Inflammatory cytokines (IL-1*β*, IL-6, TNF-*α*) In splenocytes, CPT-11 lowered the production of IL-6 (with Con A) and TNF-*α* (with Con A and anti-CD3/CD28) and of IL-1*β* (with LPS). In MLN, CPT-11 significantly increased the production of TNF-*α* (with Con A) and IL-6 (with anti-CD3/28). In splenocytes, the production of IL-6 and IL-1*β*, but not that of TNF-*α*, was normalised to levels not different from controls with Cipro; Cipro resulted in an even higher production of TNF-*α* (with LPS). In MLN, an increased production of TNF-*α* and IL-6 with CPT-11 was returned to levels not different from that of controls with Cipro.
3. Regulatory cytokine, IL-10 In the spleen, the effects of CPT-11 on IL-10 production seemed to be dependent on T-cell mitogen, with a lower Con A-stimulated production (*P*<0.05 *vs* controls), but a trend for enhancement (*P*<0.07 *vs* controls) with anti-CD3/CD28 stimulation. This effect was not altered by Cipro. In MLN, IL-10 was produced with CPT-11, after Con A stimulation, but below detection by both controls and Cipro-treated groups.
4. IFN-*γ* In splenocytes, CPT-11 lowered the production of IFN-*γ* in response to all mitogens, whereas in MLN, IFN-*γ* production was markedly higher after CPT-11, compared with that in controls (*P*<0.05). Cipro did not alter the lower production of IFN-*γ* in response to Con A and LPS, but resulted in an even lower response to anti-CD3/CD28 than CPT-11 alone. In MLN, Cipro resulted in IFN-*γ* (with Con A) levels that were lower than CPT-11 and not different from controls.

## Discussion

CPT-11 is preferentially cytotoxic to gastrointestinal (GI) mucosa, and severe diarrhoea is the hallmark toxicity for chemotherapy regimens based on this agent (Gibson *et al*, 2003; Xue *et al*, 2007). Gastrointestinal infections are particularly problematic with CPT-11, which is preferentially cytotoxic to GI mucosal cells and results in prominent GI toxicities (Gibson *et al*, 2003; Xue *et al*, 2007) at the time of profound myelosuppression. In our study, Cipro did not alter the severity or course of diarrhoea but prevented CPT-11-induced mortality. This suggests that bacteremia or septicemia secondary to CPT-11 was the major contributor to mortality in CPT-11-treated rats, and that Cipro was able to limit this by reducing the total bacterial translocation or by its immunomodulatory activity.

### Alterations of systemic and intestinal immune competence associated with CPT-11 alone

At day 7 after CPT-11 treatment, a quantitative rebound was observed in the peripheral immune compartments; blood counts of various leukocyte lineages were restored and splenic hyperplasia was present. We also observed a preponderance of activated T cells (increased percentages of CD45RA−, CD71, CD25 and decreased percentage of CD62L in CD4+ and CD8+ T cells) after CPT-11. Antigen-naive T cells are characterised phenotypically by CD45RA (high-molecular-weight isoform of CD45), and the peripheral lymph node homing receptor, CD62L (L-selectin) (Fujii *et al*, 1992; Bradley *et al*, 1994). When naive T cells are stimulated, their cell surface phenotype undergoes a number of changes. First, the expression of CD45RA is lost and CD45RO is expressed. Thereafter, CD62L is shed from cell surfaces and other markers are sequentially upregulated, including transferrin, CD71, (early) and IL-2 receptors, CD25 (late) (Lum *et al*, 1986; Jackson *et al*, 1990; Salmeron *et al*, 1995). Such a post-chemotherapy phenotypic activation has been observed earlier (Mackall *et al*, 1994; Hakim *et al*, 1997; Rutella *et al*, 2000; Wendelbo *et al*, 2004). Consistent with the expression of activation markers, immune cells from CPT-11-treated rats had increased ^3^H-thymidine uptake in the absence of mitogens and higher proportions of cells expressing IL-2 receptors. This marked *in vivo* immune activation may have been provoked by the translocation of pathogenic bacteria and their products (i.e., endotoxin).

### Hypo-responsive and anergic state of splenocytes

Quantitative changes of various cell subsets have been reported in studies on the effects of chemotherapy on the immune system; however, these changes may not reflect the functional competence of the cells. Our results show a striking discordance in phenotype and function of splenocytes after CPT-11 treatment. Despite the overall quantitative recovery and phenotypic activation of immune cells in peripheral blood and the spleen, splenocytes were unable to proliferate in response to Con A stimulation *in vitro*, and had depressed inflammatory cytokine responses to multiple mitogens. Depressed IL-2 production in response to mitogen by splenocytes is concordant with their inability to proliferate, as IL-2 production is essential for lymphocyte clonal expansion after antigen simulation. INF-*γ*, IL-1, TNF-*α* and IL-6 are also instrumental in mounting an effective inflammatory response against infection. IFN-*γ* is a pivotal cytokine initiating antimicrobial Th1 responses and plays a key role in activating macrophages and natural killer cells, whereas IL-1, IL-6 and TNF-*α* acting together are key to leukocyte transmigration, stimulating macrophage phagocytosis and evoking acute phase responses (Bendtzen, 1988; Urbaschek and Urbaschek, 1990; Van der Meide and Schellekens, 1996; Reddy *et al*, 2004). Splenocytes showed a profound inability to produce these key proliferative/inflammatory cytokines on mitogen stimulation, in addition to lacking a proliferative response, suggesting that systemic immunity (rather than local intestinal immunity) was in a state of anergy (Powell, 2006). Suppressed cell-mediated immunity could enhance the susceptibility to secondary opportunistic infection and contribute to the high mortality caused by post-chemotherapy sepsis (Ayala *et al*, 1994; O’Sullivan *et al*, 1995).

Hakim *et al* (1997) found that an *in vivo* phenotypic activation of T cells by chemotherapy was associated with a heightened susceptibility to activation-induced apoptosis on mitogen stimulation *in vitro*. Although apoptosis was not measured here, a SI with a numerical value <1.0, suggests that splenocytes were dying during the *in vitro* assay. The hypo-reactivity of spleen cells after chemotherapy could also be related to systemic infection. A similar state of hypo-responsiveness and anergy of circulating leucocytes, a phase also named as compensatory anti-inflammatory response syndrome, has been described in septic patients (Bone *et al*, 1997; Oberholzer *et al*, 2001; Hotchkiss and Karl, 2003). Taken together, we suggest that the hypo-reactivity of spleen cells after CPT-11 treatment may result from both direct immunosuppressive effects and as a consequence of systemic infection, which was likely to have been present in our animals.

### Hyper-responsive state of MLN cells

In contrast to splenocytes, cells of MLNs, a GALT compartment, showed upregulated proliferation and cytokine (i.e., IL-2, IFN-*γ*, TNF-*α*, IL-6) response to T-cell mitogens *in vitro*. Thus, the effects of CPT-11 were compartmentalised with a primed local intestinal immunity and concomitantly suppressed systemic immunity. Our results are consistent with earlier findings (de Koning *et al*, 2006) that innate and adaptive immune responses of GALT cells were intact or even primed after high-dose methotrexate. CPT-11 has been consistently shown to disrupt GI integrity, and this may expose GALT cells to bacterial antigens and LPS. Hyper-responsiveness of intestinal local immune cells is considered to contribute to chemotherapy-induced gut injury (de Koning *et al*, 2006). Antigen-driven T-cell expansion in GALT may support the homeostasis of T-cell pools after depletion by chemotherapy (Dulude *et al*, 1997). Immune hypo-reactivity in sepsis is essentially observed in peripheral blood and in the spleen (Ayala *et al*, 1993a, 1993b; Cavaillon, 2002), whereas lymphocytes derived from inflamed tissues or infectious foci are activated, primed and responsive to *in intro* mitogen stimulation (Wang *et al*, 1998; Nussler *et al*, 2001). Localisation of inflammatory response to the gut, may serve as an important strategy for preventing systemic inflammation and ignition of new inflammatory foci (Munford and Pugin, 2001).

### Effects of Cipro on alterations of immune competence after CPT-11

Antibiotics do not act solely as antimicrobial agents but also modulate innate or adaptive immune responses (Riesbeck and Forsgren, 1994; Dalhoff and Shalit, 2003). Our work is the first to systematically investigate the immunomodulatory effects of Cipro in high-dose chemotherapy, in multiple dimensions, including cell phenotype and functional competence and within distinct compartments (the spleen *vs* MLN). Overall, Cipro tended to correct splenocyte hypo-responsiveness, which may mitigate post-chemotherapy immunological anergy and favour appropriate defences against translocated pathogens; Cipro also suppressed pro-inflammatory responses occurring locally in the gut and thereby, may have a limited mucosal inflammatory injury.

A differential effect of Cipro treatment occurred in two immune tissues studied. In the spleen, Cipro did not enable a proliferative response to Con A, nor did it improve IL-2 production, but it may have prevented activation-induced cell death after CPT-11 (SI of mitogen-stimulated proliferation was raised to 1). Whether this would allow a sufficient response to blood-borne pathogens is questionable as defence against rapidly growing viral and bacterial infections requires an immediate and adequate response to limit pathogen growth and dissemination (Murtaugh and Foss, 2002).

Immunomodulatory actions of fluroquinolones rely on their ability to modify cytokine production (Dalhoff, 2005). Cipro was unable to restore IFN-*γ* production, but significantly upregulated LPS-stimulated splenocyte production of TNF-*α*, IL-1*β* and IL-6 compared with either non-Cipro-treated rats or controls. This is consistent with earlier findings with various quinolones at therapeutic levels (De Simone *et al*, 1986; Gollapudi *et al*, 1986; Bailly *et al*, 1991; Riesbeck and Forsgren, 1994; Katsuno *et al*, 2006). Cipro showed a consistent upregulation of IL-6 production of splenocytes in response to B- or T-cell mitogens, and this may be of benefit in chemotherapy-related sepsis as low Il-6 production from systemic immune compartments correlates with sepsis mortality (Adamik *et al*, 1997).

In contrast, Cipro downregulated the production of IL-2 and inflammatory cytokines by MLN cells after CPT-11 treatment, to levels observed in control rats and this is potentially important for intestinal injury. The activation and hyper-responsiveness of GALT cells contributes to the pathogenesis of chemotherapy-induced gut injury (de Koning *et al*, 2006). Excessive intestinal production of inflammatory cytokines (e.g., IL-1*β*, TNF-*α*, IFN-*γ*) is critical for developing CPT-11-related GI toxicity (Zhao *et al*, 2004).

## Figures and Tables

**Table 1:** Effects of Cipro treatment on toxicity profiles of the 3-day CPT-11 regimen at 150 mg kg^−1^ per day × 3 days

**Treatment**								
**CPT-11**	**Cipro**	***N* a**	**Mortality^b^ %**	**Incidence of severe delayed diarrhoea (%)^c^**	**Area under curve of the diarrhoea score^d^**	**Relative body weight at day 6 (%)^e^**	**Tibialis muscle gram per 1000 g body weight at day 9**	**Medial gastroncnemius muscle gram per 1000 g body weight at day 9**
None	None	8	—	—	—	98.5±0.6^f^	1.80±0.05^f^	2.05±0.02^f^
Yes	None	20	45	55.0±3.9	19.5±0.5	80.6±0.7^g^	1.60±0.03^g^	1.93±0.04^g^
Yes	Yes	11	0	47.7±5.5	18.6±0.6	90.5±0.5^h^	1.74±0.02^f^	2.04±0.04^f^

a *N*, total animal number of each treatment group.

b Mortality represents percentage of dead rats at the end of the study.

c Incidence of delayed diarrhoea was calculated for each animal by counting observations of a particular score(s) out of the total eight observations between day 3 and day 7 when diarrhoea developed to its full severity.

d Area under curve of diarrhoea score was calculated from the diarrhoea score–time graph of each individual animal between day 3 and day 7.

e Relative body weight at day 6 was calculated by comparing with the body weight at day 0.

f Means within a column that do not share a common letter are significantly different (*P*<0.05).

All data are presented as mean ±s.e.m.

f,g,h Means within a column that do not share a common letter are significantly different.

**Table 2:** Effects of CPT-11 treatment and Cipro on phenotypic distribution of immune cells in MLN and spleen

	**MLN**			**Spleen**		
**Antibody**	**Healthy controls *n*=8**	**CPT-11 alone *n*=9**	**CPT-11+Cipro *n*=6**	**Healthy controls *n*=8**	**CPT-11 alone *n*=9**	**CPT-11 + Cipro *n*=6**
B cell+(OX12) (% of total cells)	13.0±0.6^a^	19.8±1.8^b^	27.3±1.9^c^	29.1±0.4^a^	36.4±1.8^b^	36.9±1.1^b^
% OX12+CD45RA−	1.5±0.3^a^	5.3±0.6^b^	5.0±1.7^b^	7.5±0.8^a^	28.7±3.0^b^	8.3±0.9^a^
% OX12+CD45RA+	98.5±0.3^a^	94.7±0.6^b^	95.0±1.7^b^	92.5±0.8^a^	71.3±3.0^b^	91.7±0.9^a^
% OX12+CD80+	0.7±0.1^a^	1.5±0.5^a,b^	2.6±1.1^b^	2.8±0.4	3.9±0.7	2.5±0.3
CD3+(% of total cells)	70.8±1.2^a^	66.4±1.4^b^	57.5±1.2^c^	55.4±0.6^a^	46.4±2.1^b^	49.6±0.8^b^
CD3+CD8+(% of total cells)	22.2±0.8^a^	23.0±0.6^a^	17.9±0.5^b^	31.9±0.9^a^	21.0±0.9^b^	23.9±1.1^b^
% CD8+CD71+	9.2±1.2^a^	10.5±1.0^a^	19.3±2.6^b^	25.6±0.8^a^	23.9±1.2^a^	28.8±1.3^b^
% CD8+CD28+	4.0±0.6^a^	50.8±4.5^b^	75.2±2.9^c^	10.0±0.5^a^	52.3±6.3^b^	44.5±9.3^b^
% CD8+CD25+	9.3±0.3^a^	9.4±1.0^a^	40.2±9.1^b^	8.6±0.8^a^	16.4±3.7^b^	14.0±1.2^b^
% CD8+CD62 L+	38.0±2.0^a^	42.8±3.1^a^	30.1±2.4^b^	33.8±0.9^a^	34.7±3.1^a^	15.7±0.6^b^
% CD8+CD45RA−	62.4±1.7^a^	76.7±6.5^b^	38.4±2.5^c^	31.8±1.2^a^	45.9±4.6^b^	28.1±1.5^a^
% CD8+CD45RA+	37.6±1.7^a^	23.3±6.5^b^	61.6±2.5^c^	68.2±1.2^a^	54.1±4.6^b^	71.9±1.5^a^
CD3+CD4+(% of total cells)	48.7±0.7^a^	43.3±1.4^b^	40.7±1.2^b^	32.4±0.5^a^	22.2±1.0^b^	20.4±1.7^b^
% CD4+CD71+	6.5±0.7^a^	9.2±1.2^b^	4.4±0.9^a^	22.1±1.1^a^	39.9±3.8^b^	26.7±2.1^c^
% CD4+CD28+	5.5±0.3^a^	73.9±5.1^b^	85.2±2.2^b^	15.2±0.9^a^	60.7±10.4^b^	77.6±2.5^b^
% CD4+CD25+	7.5±0.4^a^	9.8±0.7^b^	37.3±11.9^c^	9.4±0.4^a^	15.4±2.2^b^	10.8±0.9^a,b^
% CD4+CD62L+	74.7±4.2^a^	53.3±6.5^b^	59.4±4.4^b^	67.8±2.1^a^	36.1±4.7^b^	28.4±1.0^b^
% CD4+CD45RA−	93.8±0.7^a^	91.9±3.4^a^	90.6±0.5^b^	80.2±0.5^a^	79.9±2.0^a,b^	83.4±1.3^b^
% CD4+CD45RA+	6.2±0.7^a^	8.1±3.4^a,b^	9.4±0.5^b^	19.8±0.5^a^	20.1±2.0^a,b^	16.6±1.3^b^
CD3+CD4+/CD3+CD8+	2.2±0.1^a^	1.9±0.0^b^	2.3±0.1^a^	1.0±0.0^a^	1.1±0.0^a^	0.9±0.1^b^
CD71 (% of total cells)	15.8±0.7^a^	12.8±0.9^b^	14.6±0.6^a,b^	23.5±0.8^a^	33.5±2.6^b^	22.6±2.1^a^
CD25 (% of total cells)	4.3±0.2^a^	5.7±0.4^b^	27.1±7.5^c^	5.5±0.5^a^	10.2±1.9^b^	6.5±0.6^a,b^
CD28 (% of total cells)	3.4±0.2^a^	44.8±3.2^b^	55.4±1.9^c^	7.4±0.5^a^	29.3±2.9^b^	38.5±3.5^b^
CD62 L (% of total cells)	48.2±2.8^a^	46.7±2.5^a^	35.3±1.1^b^	39.3±1.2^a^	31.4±1.4^b^	15.3±0.5^c^
CD45RA+(% of total cells)	36.3±1.3^a^	24.8±1.7^b^	35.0±1.6^a^	56.3±0.5^a^	36.4±3.2^b^	52.0±1.1^c^

a Data presented as cell population percentage mean±s.e.m.; means within a row for a given cell type (splenocytes, mesenteric lymph node (MLN) cells) that do not share a common letter are significantly different (*P*<0.05). ^a,b,c^Means within a column that do not share a common letter are significantly different.

**Table 3:** Effects of CPT-11 treatment and Cipro on *in vitro* proliferation in response to Con A by immune cells in MLN and spleen

		**MLN**			**Spleen**		
**Mitogen**	**Unit**	**Healthy controls**	**CPT-11 alone**	**CPT-11+Cipro**	**Healthy controls**	**CPT-11 alone**	**CPT-11+Cipro**
*24 hours*							
None	DPM	668±51 (8)^a^	1359±143 (9)^b^	482±65 (8)^c^	2954±75 (8)^a^	11204±1046 (9)^b^	3498±348 (8)^a^
							
*48 hours after mitogen stimulation*							
None	DPM	317±53 (8)^a^	1023±128 (5)^b^	251±57 (7)^a^	5001±588 (8)^a^	5603±840 (9)^a^	3789±778 (8)^a^
CON A	SI	35±10 (8)^a^	104±16 (5)^b^	29±11 (7)^a^	8.70±1.50 (8)^a^	0.10±0.02 (9)^b^	1.05±0.33 (8)^c^

SI=stimulation index.

a Data are presented as mean±s.e.m. (number of rats), means within a row for a given cell type (splenocytes, mesenteric lymph node (MLN) cells) that do not share a common letter are significantly different (*P*<0.05).

b Simulation index. ^a,b,c^Means within a column that do not share a common letter are significantly different.

**Table 4:** Effects of CPT-11 treatment with our without Cipro on mitogen-stimulated cytokine production by splenocytes and MLN cells

**Mitogen**	**Cytokine ( × 10^−9^ g l^−1^)**	**Healthy controls**	**CPT-11 alone**	**CPT-11+ Cipro**
*Spleen*				
Con A	IL-2	5020±76 (4)^a^	2646±234 (9)^b^	2398±475 (6)^b^
	IL-6	620±19 (4)^a^	329±24 (9)^b^	535±32 (6)^a^
	IL-10	581±38 (4)^a^	194±19 (9)^b^	181±29 (6)^b^
	TNF-*α*	930±23 (4)^a^	257±26 (9)^b^	197±20 (5)^b^
	IFN-*γ*	3637±190 (4)^a^	1663±157 (9)^b^	1185±260 (6)^b^
				
Anti-CD3/28	IL-2	502±37 (4)^a^	227±21 (9)^b^	199±73 (5)^b^
	IL-6	514±140 (4)^a,b^	303±45 (7)^b^	449±25 (5)^a^
	IL-10	129±60 (4)^b^	248±31 (9)^a,b^	355±60 (5)^a^
	TNF-*α*	210±12 (4)^a^	95±14 (9)^b^	100±28 (5)^b^
	IFN-*γ*	1972±386 (4)^c^	683±128 (9)^a^	149±50 (5)^b^
				
LPS	IL-1*β*	252±24 (4)^a^	134±11 (9)^b^	196±19 (7)^a^
	IL-6	880±82 (4)^a,b^	868±87 (9)^b^	1247±157 (7)^a^
	TNF-*α*	372±16 (4)^b^	437±56 (9)^a,b^	577±64 (7)^a^
	IFN-*γ*	1958±160 (4)^a^	244±75 (9)^b^	127±42 (7)^b^
				
*MLN*				
Con A	IL-2	908±113 (4)^b^	2882±364 (6)^a^	1381±264 (6)^b^
	IL-6	137±10 (4)	129±28 (8)	117±16 (7)
	IL-10	—^*^	67±22 (6)	—^*^
	TNF-α	68±11 (4)^b^	592±83 (8)^a^	138±26 (6)^b^
	IFN-*γ*	371±51 (4)^b^	2551±506 (7)^a^	404±131 (6)^b^
Anti-CD3/28	IL-6	23±4 (2)^b^	176±19 (8)^a^	39±9 (5)^b^
	IL-10	94±34 (3)	66±16 (8)	—^*^

^*^— below detection limit.

^a^Data are presented as mean±s.e.m. (number of rats), means within a row that do not share a common letter are significantly different (*P*<0.05). ^a,b^Means within a column that do not share a common letter are significantly different.
